# Determining Factors for Stress Perception Assessed with the Perceived Stress Scale (PSS-4) in Spanish and Other European Samples

**DOI:** 10.3389/fpsyg.2018.00037

**Published:** 2018-01-26

**Authors:** Miguel A. Vallejo, Laura Vallejo-Slocker, Enrique G. Fernández-Abascal, Guillermo Mañanes

**Affiliations:** Faculty of Psychology, Universidad Nacional de Educación a Distancia, Madrid, Spain

**Keywords:** stress, perceived perception stress, normative values, psychometric, cross-cultural assessment

## Abstract

**Objective:** Stress perception depends on cultural and social aspects that vary from one country to another. One of the most widely disseminated methods of assessing psychological stress is the Perceived Stress Scale (PSS-4). Therefore, in order to identify these factors and their impact on mental health, the present study compares the PSS-4 results among three European countries (Great Britain, France and Spain). This study focuses on PSS-4 results within a Spanish sample to determine: (1) normative data, reliability and validity of PSS-4 in a Spanish sample and (2) how stress perception changes depending on cultural and social factors.

**Methods:** The data were obtained from a website representing a service of a smoking cessation program, the study represented a service that was open to all individuals. The number of participants were 37,451. They reported their age, gender, nationality, marital status, education and employment status, and completed two psychological questionnaires (PPS-4 and the anxiety and depression scales of the Symptom Checklist-90-Revised, SCL 90-R).

**Results:** The PSS-4 scores could differentiate between relevant sociodemographic variables (such as sex, age, nationality, marital status, education, parental status, employment status, and income class). The PSS-4 scores showed a positive correlation with the SCL 90-R anxiety and depression scales. The normed values for interpreting the PSS-4 scores are presented. The PSS-4 showed adequate internal consistency and reliability.

**Conclusions:** The PSS-4 is a useful instrument for assessing stress perception levels in the general population in different countries. Its internal consistency is sufficient for a 4-item scale.

## Introduction

Stress is an important reference point in health studies and it is related to both an individual's general health status and different illnesses, including mental disorders, cancer, cardiovascular disease, drug abuse, chronic diseases, etc. Since stress is a cross-cultural symptom for many different types of health problems, understanding stress across different sociodemographic, cultural and social groups could help to prevent stress-related problems and major health concerns worldwide. The prevalence of mental disorders in Europe shows the importance of considering stress variables. Key reasons include mental disorders frequently being observed in females (Dedovic et al., 2009), the unemployed, persons who never married, youngers or countries where their living (ESEMeD, 2004). The cultural factors, including the social support, are fundamental to understanding how the people perceive stress and how they cope with it (Kim et al., 2008). These differences are present in European countries as UK, Germany, France, Netherlands, Spain and Belgium and show how the effect of the stress is different (Cao et al., 2016). Thus, evaluating the way in which individuals perceive stressful situations in their lives is critical for the quantification of psychological stress in health and disease worldwide. In particular, this research is necessary for understanding, preventing, and treating many health problems beyond national boundaries.

One of the most widely disseminated methods of assessing psychological stress has been the Perceived Stress Scale (PSS; Cohen et al., 1983). This self-report scale generates a global stress score that is based on general questions rather than focusing on specific experiences. This scale could be useful to compare stress perception in different countries. With this scale, subjects are asked to evaluate the previous month before the time of the self-report. The PSS was originally a 14-item scale, although the scale was reduced to 10 items (Cohen and Williamson, 1988) when 4 poorly performing items were identified and removed. Additionally, this was further reduced to 4 items for use in situations in which measurements must be obtained quickly. The PSS-4 scale has a clear advantage in terms of the time required to complete and ease of use, and this assessment is easy to complete on the Internet (Herrero and Meneses, 2006; Mañanes et al., 2016). The main reason for choosing the PSS-10 instead of the PSS-4 is not reliability (Cronbach α), as several studies have shown a reliability level of α = 0.67 for the PSS-10 (Leung et al., 2010) versus α = 0.82 for the PSS-4 (Mitchell et al., 2008). Although the PSS-4 shows better internal consistency than the PSS-10, the magnitude of the difference is more dependent on the study characteristics as opposed to the PSS scale (Lee, 2012).

These PSS scales have been studied, translated and adapted to several languages and countries, which on one hand demonstrates the relevance of this instrument and on the other hand, demonstrates the importance of stress among different societies. Warttig et al. (2013) published normative data for the PSS-4 in an English-speaking sample (population of several Primary Care Trusts from England and Wales), and Lesage et al. (2012) provided similar data from a French sample (workers selected from several occupational health care centers of the North of France). These authors published the norms in means and standard deviation (SD) for gender, group ages, ethnicity and other relevant variables. Additionally, these data allow for the comparison of different studies and samples. Lee (2012) reviewed the psychometrical data in 19 studies that used the PSS-14, PSS-10, and PSS-4 scales. He concluded that the PSS scales show acceptable psychometric properties, albeit with differences related to population diversity, such as gender, age, parental status and other sociodemographic variables. Other studies (Cohen and Williamson, 1988; Herrero and Meneses, 2006; Karam et al., 2012; Lee et al., 2015; Ingram et al., 2016) used PSS-4 but did not offer norm values. Nevertheless, they informed of mean and standard deviation of a general sample, male, and female. It is useful to compare these data with the data obtained in normative studies.

Thus, it is necessary to evaluate these scales further. Particularly to obtain norms from broad representative samples and understand stress among cultures.

Within Europe, the PSS-4 has been tested among British and French samples. However, there are no studies of this scale in Spanish speaking countries. The PSS-4 may be useful for broadening knowledge about the determinants of stress because of its shortness, stress approach, Internet suitability and psychometric properties. Thus, the aim of this study was to examine a broad sample of the Spanish population to determine the following: (1) psychometric characteristics of the PSS-4 in terms of: normative data, reliability and validity in a Spanish sample and (2) how stress perception changes depending on cultural and social factors by comparing the results of this study with the British and French samples (a reference is made in relation to a Canadian and Korean study about stress assessed with PSS-4).

## Methods

The data for this study were obtained from the smoking cessation program website of the Universidad Nacional de Educación a Distancia (UNED) (https://www.apsiol.uned.es/dejardefumar) from October 2009 to November 2014. The study represented a service that was open to all individuals. The Bioethical Committee of the UNED approved the study. Before initiating the data introduction, informed consent was obtained from all participants.

A total of 37,451 people were interested in participating. Those who were interested in enrolling in the study were required to submit sociodemographic data, including age, gender, nationality, marital status, education and employment status, as well as two psychological questionnaires: the PPS-4 (Cohen et al., 1983; Cohen and Williamson, 1988) and the anxiety and depression scales of the Symptom Checklist-90-Revised (SCL 90-R) (Derogatis, 1977; González de Rivera et al., 1989). Additional details about the study have been published elsewhere (Mañanes and Vallejo, 2014; Mañanes et al., 2016). All the data were obtained from the Web application before the smoking cessation program began. There was not a selection of data. All the data registered was accepted.

The PSS-4, as well as, the SCL 90-R had already been tested in Spanish speaking samples in on-line interventions. We used the PSS-4 adapted by Herrero and Meneses (2006). The scale has four items in only one scale. These four items were: in the last month,

1. how often have you felt that you were unable to control the important things in your life?
2. how often have you felt confident about your ability to handle your personal problems?
3. how often have you felt that things were going your way?
4. how often have you felt difficulties were piling up so high that you could not overcome them?

A high score indicates a high perception of stress. The Cronbach's α coefficient = 0.72; and a Principal Component analyses show one factor that explains a 54% of the variance. Herrero and Meneses (2006) used a point scale ranging from 1 to 5 instead of the original scale used by Cohen (0–4). We used Cohen's original scale (0, never; 1, almost never; 2, sometimes; 3, fairly often; 4, very often) to score the 4-item scale. The SCL 90-R was also presented in Spanish on the Internet with a Cronbach's α coefficient = 0.97 (Vallejo et al., 2008).

The broad sample of people included in the study allowed us to examine the utility and psychometric structure of the PSS-4 in a Spanish population and to compare these data with other studies that have provided desegregated data from this questionnaire. An alpha level of 0.05 was used for the statistical test. An analysis of variance (ANOVA) was used to analyze the differences between the subgroups of the sample. A *t*-test was used to compare the results of this study with other studies when appropriate. The Pearson correlation coefficient was used to relate the quantitative variables.

To evaluate the PSS-4 psychometric properties, a reliability analysis was performed. We calculated the Cronbach's α coefficient, the Spearman Brown split-half reliability coefficient, and the standard error of measurement (SEM). A validity analysis included a correlation between the PSS-4 scores and the anxiety and depression SCL 90-R scores as well as the differences between the PSS-4 scores and the different relevant variables.

We performed a Principal Component analysis to determine the PSS-4 structure. We selected an Oblimin rotation as proposed by Lesage et al. (2012). Eigenvalues above 1 were retained.

## Results

### Sample description

The analysis of the sample (*n* = 37,451) showed a distribution similar to the Spanish population, as obtained through the Spain Census. Table 1 shows the characteristics of the sample, which comprised 47.2% male and 52.1% female subjects. The mean age in the sample (38.9 years) was very close to that of the Spanish population (38.8 years). In the sample, 91.1% of the subjects were Spanish, similar to the frequency of 90.5% in the general population; 1.7% represented citizens of other EU countries, and 7.2% were from outside the EU, likely, for language reasons, from Spanish-speaking countries. The marital status of the sample showed that 43.8% were married and 35.8% were single. In terms of education, 47.3% completed university studies, 18.9% had professional training and 20.9% had high school education; the remaining 12.9% had primary-level education. In terms of employment, 75.9% were currently working and 24.1% were unemployed. This distribution fits perfectly with that reported in the Spain Census. Finally, in terms of income, 62.3% belonged to the middle class, with a normal curve distribution showing slow predominant movement to the lower socioeconomic levels.

**Table 1:** Sample characteristics.

	**Total**	**N**	**%**	**Spain census 2014 %**
Sex	Male	17,673	47.2	49.1
	Female	19,778	52.1	50.9
Age	Years	Mean = 38.9	*SD* = 10.3	Mean = 38.8
Nationality	Spanish	34,116	91.1	90.5
	Other EU	629	1.7	4.2
	Non-EU	7,541	7.2	5.3
Marital status	Single	13,393	35.8	
	Married	16,403	43.8	
	Separated	3,425	9.2	
	Living with partner	3,870	10.3	
	Widowed	360	0.9	
Education	Primary	4,844	12.9	
	High school	7,818	20.9	
	Professional training	7,070	18.9	
	University	17,719	47.3	
Employment status	Working	28,512	75.9	75.7
	Unemployed	8,939	24.1	24.3
Income class	Upper	223	0.6	
	Upper-middle	4,343	11.6	
	Middle	23,330	62.3	
	Lower-middle	7,241	19.3	
	Bottom	2,314	6.2	

*N = 37,451. EU, European Union*.

### PSS-4 scale analysis

Table 2 shows the PSS-4 item scores with the mean and standard deviation. The mean score for the PSS-4 was 5.43 with a SD of 2.96. The kurtosis and skewness values indicated a normal distribution: below 0.05 in the global scale and below 0.1 for each item.

**Table 2:** Means, standard deviations, kurtosis and skewness for items and total scores of the PSS-4.

**PSS-4**	**Mean**	***SD***	**Kurtosis**	**Skewness**
Item 1	1.17	1.02	−0.71	0.65
Item 2	1.36	1.04	−0.45	0.64
Item 3	1.55	0.88	−0.23	0.23
Item 4	1.35	1.02	−0.94	0.56
Total score	5.43	2.96	−0.14	0.41

*N = 37,451*.

The internal consistency of the scale was 0.74 (Cronbach's α). The Spearman-Brown split-half reliability coefficient was 0.76, and SEM was 1.53. According to this SEM, the interval indicating the true score was 5.34 ± 1.53 (3.81–6.87).

The Kaiser-Meyer-Olkin (KMO = 0.794) index from the exploratory factor analysis confirmed that the sampling is adequate. The analysis obtained only one factor, explaining 56.4% of the total variance. The factor loadings were 0.78, 0.63, 0.79, and 0.81 for items 1–4, respectively. Table 3 shows the factor loadings for principal component analysis.

**Table 3:** Factor loadings for principal component analysis.

**PSS-4**	**Communality**	**Factor loading**
**ITEM 1**		
Unable to control the important things in your life	0.60	0.78
**ITEM 2**		
Confident about your ability to handle your personal problems	0.50	0.63
**ITEM 3**		
Things were going your way	0.61	0.79
**ITEM 4**		
Difficulties were piling up so high that you could not overcome them	0.65	0.81

*N = 37,451. Eigenvalue = 2.26. Percentage of variance explained = 56.4%*.

The PSS-4 scores showed a positive correlation with the SCL 90-R anxiety and depression scales: *r* = 0.51 and *r* = 0.69, respectively. The correlations were significant (*p* < 0.05) and indicated concurrent validity with the PSS-4. A difference in stress perception was identified between working and unemployed subjects: 5.16 vs. 6.33; *F* = 10871.31 (1, 37449), with *p* < 0.001. Other differences between the groups with known distinct levels of stress, such as gender, age, education, parental status or income class, are shown in Table 4. The PSS-4 was able to differentiate between these groups.

**Table 4:** Norms for the stratified scores of the PSS-4.

		**Present study**						**French study (Lesage et al., 2012)**^¥^				**British study (Warttig et al., 2013)**			
		***n***	***M***	***SD***	**Q1**^§^	**Q3**^§^	**F(fd)**	***n***	***M***	***SD***	**t(fd)**	***n***	***M***	***SD***	**t(fd)**
Total		37,451	5.43	2.95	3	7		501	5.40	2.90	0.23 (35950)	1,484	6.11	3.14	10,37 (38933)^***^
Sex	Male	17,673	5.25	2.91	3	7	132,19 (1, 37449)^***^	249	5.20	2.50	0.31 (17920)	491	5.56	3.04	2.23 (18162)
	Female	19,778	5.60	2.99	3	8		252	5.60	3.20	0.00 (20028)	993	6.38	3.15	7.63 (20769)^***^
Age (years)	<18	229	6.25	2.95	4	8	24.16 (6, 37449)^***^	–	–	–	–	22	6.91	2.89	1.02 (249)
	18–29	7,180	5.73	2.98	4	8		125	4.70	2.00	5.65 (7303)^***^	409	6.66	3.23	5.68 (7587)^***^
	30–44	18,816	5.38	2.94	3	7		133	5.30	2.60	0,35 (18947)	596	6.05	3.16	5.11 (19410)^***^
	45–54	8,547	5.38	2.94	3	7		129	5.90	3.20	1.83 (8584)	264	5.69	3.04	1.63 (8809)
	55–64	2,368	5.08	2.93	3	7		114	5.60	2.40	2.23 (2480)	143	5.76	2.87	2.75 (2509)
	>65	311	4.88	2.90	3	7		–	–	–	–	50	5.32	2.86	1.01 (359)
Nationality	Spanish	34,116	5.38	2.94	3	7	52.16 (2, 37449)^***^	501	5.40	2.90	0.15 (34615)	1,484	6.11	3.14	8.79 (35598)^***^
	Other EU	629	6.05	2.95	4	8		501	5.40	2.90	3.71 (1128)^***^	1,484	6.11	3.14	0.42 (2111)
	Non-EU	7,541	5.9	3.08	4	8		501	5.40	2.90	3.72 (8040)^***^	1,484	6.11	3.14	2.36 (9023)
Marital status	Single	13,393	5.69	2.96	4	8	85.28 (4, 37449)^***^	162	5.50	3.20	0.75 (13553)				
	Married	16,403	5.13	2.89	3	7		337	5.30	2.70	1.14 (16738)				
	Separated	3,425	5.85	3.09	4	8		–	–	–	–				
	LWP	3,870	5.45	2.96	3	7		–	–	–	–				
	Widowed	360	5.51	2.99	3	7		–	–	–	–				
Education	Primary	4,844	6.20	3.12	4	8	1684.26 (3, 37449)^***^	176	5.80	2.90	1.79 (5018)				
	HS	7,818	5.62	2.99		8		149	5.10	3.10	2.07 (7965)				
	PT	7,070	5.52	2.86	3	7		53	5.50	2.30	0.06 (7121)				
	University	17,719	5.11	2.88	3	7		123	5.10	2.60	0.04 (17840)				
Parental status	C	19,758	5.32	2.96	3	8	63.01 (1, 37449)^***^	335	5.60	2.80	1.81 (20089)				
	NC	17,692	5.56	2.94	3	7		166	4.90	3.00	2.82 (17856)^***^				
Employment status	Working	28,512	5.16	2.85	3	7	1097.31 (1, 37449)^***^								
	Unemployed	8,939	6.33	3.12	4	8									
Income class	Upper	223	3.82	2.86	2	5	591.75 (4, 37449)^***^								
	Upper-middle	4,343	4.50	2.84	2	6									
	Middle	23,330	5.18	2.80	3	7									
	Lower-middle	7,241	6.24	2.97	4	8									
	Bottom	2,314	7.40	3.24	5	10									

*** *p > 0.001*,

**p > 0.01, *p > 0.05, Bonferroni adjusted for multiple comparisons, LWP, living with partner; PT, professional training; HS, high school; C, with children; NC, without children.

¥ *The age intervals and the education/occupational statuses were adapted to allow for comparisons*.

§ *A score equal or lower than Q1 value indicates that this person belongs to the group of people with lower stress perception. He belongs to 25% of the people with lower stress perception. A score equal or higher than Q3 value indicates that this person belongs to the group of people with higher stress perception. He belongs to 75% of the people with higher stress perception*.

### Norm values

Table 4 shows the disaggregated data of the PSS-4 scores for the different groups that were identified within the sample. The groups included sex (male, female), age intervals, nationality (Spanish, other European Union countries (EU), non-EU), marital status (single, separated, living with partner, widowed), education level (primary, high school, professional training, university), parental status (with children, without children), employment status (working, unemployed) and income class (upper, upper-middle, middle, lower-middle, lower). The number of subjects with the mean, standard deviation and quartile 1 and 3 values are shown for each group.

Table 5 shows other studies which used PSS-4 and do not offer norm values. Nevertheless, they informed of mean and standard deviation of a general sample, male, and female. It is useful to compare these results with those obtained in our study.

**Table 5:** PSS-4 scores in different studies.

		***N***	***M***	***SD***	**Scale**	**Range**	***M* (0–16)**
Present study	Total	37,451	5.43	2.95	0–4	0–16	5.43
	Male	17,673	5.25	2.91	0–4	0–16	5.25
	Female	19,778	5.60	2.99	0–4	0–16	5.60
Ingram et al., 2016^a^	Total	225	11.05	3.41	1–5	4–20	7.05^*^
	Male	135	10.77	3.59	1–5	4–20	7.77^*^
	Female	90	11.44	3.23	1–5	4–20	8.44^*^
Karam et al., 2012^b^	Female	217	2.88	2.93	0–4	0–16	2.88^*^
Herrero and Meneses, 2006^c^	Internet	262	9.25	2.37	1–5	4–20	5.25
Lee et al., 2015^d^	Male	159	6.27	2.13	0–4	0–16	6.27^*^
	Female	243	7.22	2.41			7.22^*^
Cohen and Williamson, 1988^e^	Male	946	4.20	2.80	0–4	0–16	4.20^*^
	Female	1,784	4.70	3.10			4.70^*^

* p > 0.001;

a USA, Low socioeconomic;

b USA and Canada, Pregnant;

c Spain;

d Korea;

e *USA*.

## Discussion

To our knowledge, this study used the largest population sample to study the PSS-4. Our results provide the opportunity to (1) analyze how stress is perceived throughout different cultures by comparing our results with other studies; (2) use this scale in a wide sample of Spanish (one of the most widely spoken languages) obtaining new norms for interpreting the scores; and (3) add information about the psychometric characteristics of this instrument.

### Mean

We obtained a mean of 5.43, which was identical to the result from the study by Lesage et al. (2012), which used the French version of the PSS-4 (see Table 4). Several differences exist between other studies. In the British study (Warttig et al., 2013), the mean was 6.11. This result was statistically different (see Table 4) and may have been due to the comparison between Spanish and British participants. It actually seems like the higher differences are between British and other EU nationals (Spanish and French people have lower PSS-4 scores, 5.4, than British people, 6.1). Moreover, when people in our study with other EU nationalities were compared to the British sample, the difference was not significant. It is possible that, in relation to stress, people with other EU nationalities in Spain (6.05) share common characteristics with British (6.11) thus making them respond similarly to British and differently from Spanish (5.38) and French (5.40) people.

Other studies have also reported different means for stress perception. Table 5 shows these data with a correction when the original 0–4 scale was not used. Ingram et al. (2016) obtained higher scores (7.05), which may have been due to a lower socioeconomic level of the participants. In our study (see Table 4), scores increased with both lower income classes (7.4 for the bottom income class) and lower education levels (6.02 for primary level education). Lee et al. (2015) also obtained a higher mean (6.27) than could be explained due to cultural factors (Korean sample). A low mean (2.88) was presented by Karam et al. (2012), but this represented a specific population of pregnant women. The other two studies included in Table 4 reported values near the 5.4 mean of our study and the French study. In the study by Herrero and Meneses (2006), there was no significant statistical difference, and in the case of the original study by Cohen and Williamson (1988), a small difference was identified.

In summary, the mean of 5.4 obtained in our study may constitute a valid reference for perceived stress in Spain. When the mean is considered in the interval that is limited by the SEM (3.81–6.87), all the means can be included, with the exception of those with specific values (income class, education, etc.). Table 4 includes two columns with information about quartiles 1 and 3 to help with the interpretation of the data in each subsample.

### Age

Stress scores showed a tendency to decrease with age, from 6.25 in ages <18 years to 4.88 in ages >65 years. Similar findings were obtained by Cohen and Williamson (1988) and Warttig et al. (2013). This trend was not apparent in the French study (Lesage et al., 2012), although this difference may have been due to the different intervals used to classify these variables. Several theories offer reasons for this decline of stress with age, from the selectivity of positive aspects to reduced physical reactivity due to physical and health limitations (Carstensen et al., 1999, 2006).

### Nationality

Nationality is one way to consider cultural differences, which may be why the French and Spanish scores are more similar to one another than the British scores. In our study, the lower scores were provided by Spanish people (5.38), and these scores increased (5.9) when the subjects were Spanish-speaking (non-EU citizens) or had other EU nationalities (6.05) (likely immigrants). A similar increase was shown in the study by Warttig et al. (2013) related to ethnicity, including Caucasians compared to African-American, mixed and Asian subjects (with the exception of Chinese participants). The sensitivity of the PSS-4 to these cultural and ethnicity factors is a positive aspect of the scale and supports the results of other studies (Geronimus et al., 2006). Being able to assess stress patterns within a particular cultural environment is necessary to understand the nature of stress.

### Marital status

The lowest PSS-4 scores were obtained for married subjects (5.13) while the highest were obtained by separated subjects (5.85). The distribution obtained in our study showed that living with a partner had a positive effect on reducing stress. The same trend was found in the French study. Single and widowed individuals were in the middle of the score range. The protection/support hypothesis in favor of married individuals has been supported by various studies (Coombs and Fawzy, 1982). Additionally, population data show poor mental health for unmarried and widowed individuals compared to married individuals or those living with partners (Lindström and Rosvall, 2012).

### Education

In terms of education, subjects with the lowest education identified the highest amount of stress (6.20) while those with the highest about of education seem to identify the least (5.11). Lesage et al. (2012) obtained a similar result (5.80–5.10, respectively) while subjects with professional training or a High School education scored very similarly (5.52 and 5.62, respectively). Comparing the educational level of participants across different countries is a difficult task. The role of the educational level has not been acknowledged in the stress bibliography when considering the general population. Fiocco et al. (2007) found that people with lower levels of education were more stressed in reaction to the Trier Social Stress Test than individuals with higher levels of education. Instead of focusing on the importance of the education level, other variables, such as social or parental support, may mediate the education level influence on stress (Parkes et al., 2015).

### Parental status

Parental status is another familiar factor that may be related to stress. In our study, people with children showed low (5.32) PSS-4 scores compared to individuals without children (5.56). These data suggest that having children is a protective factor against stress. However, the French study (Lesage et al., 2012) showed a different result, in which subjects with children obtained a higher PSS-4 score (5.60) than those without children (4.90). In this case, having children was identified as a vulnerability factor for stress, and a similar result was obtained in Canada (Muhammad and Gagnon, 2009). The difference observed in our study may be explained in sociocultural terms, as it is possible that the social support of an extended family has a protective role in stress perception.

### Employment status

Unemployed subjects showed higher PSS-4 scores than employed individuals. This effect is congruent with many studies related to this issue. Economic and noneconomic factors are likely responsible for this difference (Frasquilho et al., 2016), and there is a clear independent effect of unemployment on mental distress (Backhans and Hemmingsson, 2012).

### Income class

The PSS-4 scores clearly reflected the six income classes defined in our study, from the lower class (7.40) to the upper class (3.82). These results confirmed previous studies showing a close relationship between stress and low income. Indeed, having a low income is a predictor of a variety of psychological problems (DeCarlo Santiago et al., 2011), and the perception of stress overlays other variables, such as gender or age (Panjwani et al., 2016).

The validity of the PSS-4 was confirmed in our study by the positive correlation with depression and anxiety scales of SCL 90-R, although the principal reason for recognizing the validity of this questionnaire is the power to differentiate between groups that are under different levels of stress. The internal consistency of the PSS-4 was similar to the values reported by other studies. Our alpha of 0.74 is within the range of those found by Cohen and Williamson (0.60, 1988) and Mitchell et al. (0.82, 2008). The Spearman Brown split-half reliability coefficient obtained was close to that obtained by Mitchell et al. (2008), and the SEM was slightly lower.

The factor structure obtained through the principal component analysis supports the one factor solution of the PSS-4. Similar results were obtained by others (Cohen and Williamson, 1988; Mitchell et al., 2008; Lesage et al., 2012), but others have questioned whether these four items really load onto two factors (Ingram et al., 2016).

In summary, our results show that the PSS-4 is useful for assessing stress in the general population in Spain and to establish comparisons with different countries. Our study, using a sample of 37,451 subjects, enabled the differentiation and facilitation of norms between subjects according to several sociodemographic variables. The results obtained are close to those obtained with other samples. Although cultural factors are important, the effect in different contexts exists when socioeconomic factors are considered. To this extent, not being able to compare all categories defined in our study to equivalent data in other samples was considered a limitation for the analysis of stress across cultures. Finally, the internal consistency of the PSS-4 is sufficient for a 4-item scale and for gathering information on the perception of stress.

## Ethics approval and consent to participate

The study protocol was approved by the Bioethical Committee of the UNED. All participants gave written informed consent when they entered the study.

## Author contributions

MV and GM designed the study and the aplication web needed to performing it. LV-S and EF-A constructed the conceptual framework of the work. MV and LV-S performing the data retrival and statistical analysis and preparing the initial draft of the manuscript. All authors have read and approved the final version of the manuscript.

### Conflict of interest statement

The authors declare that the research was conducted in the absence of any commercial or financial relationships that could be construed as a potential conflict of interest.

## Abbreviations

PSS-4: Perceived Stress Scale 4 items
SD: Standard Deviation
SEM: Standard Error of Measurement (SEM)
SCL 90-R: Symptom Checklist-90-Revised.
